# Treatment Adherence and Health Status of Patients With COPD Under Treatment With Salmeterol/Fluticasone via the Elpenhaler® Device: The AHEAD Study

**DOI:** 10.1111/crj.13803

**Published:** 2024-07-26

**Authors:** Konstantinos P. Exarchos, Georgios Hillas, Paschalis Steiropoulos, Polyanthi Papanastasiou, Athena Gogali, Konstantinos Kostikas

**Affiliations:** ^1^ Respiratory Medicine Department University of Ioannina School of Medicine Ioannina Greece; ^2^ 5th Pulmonary Department “Sotiria” Chest Diseases Hospital Athens Greece; ^3^ Department of Respiratory Medicine, Medical School Democritus University of Thrace Alexandroupolis Greece; ^4^ Medical Department ELPEN Pharmaceutical Co. Inc. Athens Greece

**Keywords:** adherence, chronic obstructive pulmonary disease, COPD

## Abstract

**Background:**

Chronic obstructive pulmonary disease (COPD) is a heterogeneous progressive lung condition characterized by long‐term respiratory symptoms and airflow limitation. Appropriate bronchodilation is the cornerstone of COPD treatment, leading to better health status as well as benefits in prognosis and mortality.

**Methods:**

In the current open, noninterventional, observational study, 716 patients diagnosed with COPD of variable severity were administered a fixed‐dose combination (FDC) of fluticasone propionate and salmeterol (500 + 50 mcg) through the Elpenhaler® device. The patients' adherence to treatment (based on the MMAS‐8 [8‐item Morisky Medication Adherence Scale]) and health status (based on the CCQ [Clinical COPD Questionnaire]) were assessed at the beginning of the study and at the end of the 3‐month follow‐up period.

**Results:**

The mean ± SD MMAS‐8 score at 1 and 3 months was 6.12 ± 1.89 and 6.45 ± 1.80, respectively, indicating medium adherence overall; however, there was a statistically significant increase of 0.33 units in the MMAS‐8 score at the end of the follow‐up (paired *t*‐test *p* < 0.0001), suggestive of an improvement in adherence throughout the study. Higher adherence was associated with better health status at baseline, which further improved by the end of the follow‐up. Moreover, we observed a statistically significant decrease of 1.07 points (*p* < 0.0001) in the mean CCQ total score from the baseline (CCQ score = 2.2 ± 1.00) until the end of the study follow‐up (CCQ score = 1.13 ± 0.67). Similar conclusions were also drawn in the mean domain scores regarding symptoms (score equal to 1.36 ± 0.72, decrease by 1.18) as well as functional and mental state (scores equal to 0.86 ± 0.73 and 1.20 ± 0.88, decrease by 1.04 and 1.00, respectively, *p* < 0.0001). Similarly, when patients were stratified into subgroups with and without comorbidities, the former group showed an increase of 7% in the patients with medium to high adherence during the course of the study. In the same patient subgroup, there was a notable decrease in CCQ score by 1.18 points (*p* < 0.0001) during the study.

**Conclusions:**

The administration of FDC of fluticasone propionate and salmeterol, (500 + 50 mcg) via the Elpenhaler® device for COPD, resulted in a well‐maintained or slight increase in treatment adherence and a subsequent benefit in health status, which further persisted after 3 months of treatment.

## Graphical abstract

A study of 716 COPD patients found that the combination of fluticasone propionate and salmeterol, administered through the Elpenhaler® device, improved treatment adherence and health status over a 3‐month period. This suggests that this treatment strategy is effective for managing COPD.

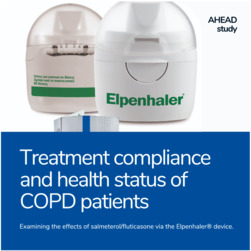

## 1 Introduction

Chronic obstructive pulmonary disease (COPD) is a preventable and treatable disease affecting a significant portion of the population (approximate global prevalence of 11.7%), inducing significant morbidity and mortality [1]. COPD is characterized by diffuse small airway inflammation and the destruction of lung parenchyma, resulting in persistent and progressive airflow limitation. The most common respiratory symptoms include cough with or without sputum production and dyspnea; fatigue, anorexia, and weight loss can arise as the disease progresses [2]. The most prominent risk factor is cigarette smoking as well as exposure to other noxious particles, e.g., air pollution. COPD is the outcome of the often complex interplay between external exposure and host factors such as genetic abnormalities, poor lung growth during childhood, and accelerated aging. The natural course of the disease is marked by periods of acute worsening of respiratory symptoms, called exacerbations. COPD exacerbations call for additional therapy and are often attributed to infectious causes.

The treatment of stable COPD is largely decided based on clinical criteria encompassing exacerbation history and symptom severity [3]. Though inhaled corticosteroids (ICSs) alone are not favored for the treatment of stable COPD, the combination of bronchodilators including ICS has been extensively prescribed. It should be noted that according to the latest GOLD report [3], the use of ICS + LABA (long‐acting β2‐receptor agonists) is no longer encouraged, and in cases where ICS is indicated, triple therapy, i.e., ICS + LABA + LAMA (long‐acting muscarinic antagonists), should be preferred. Nevertheless, stable patients already treated with ICS + LABA could maintain this treatment combination [4]. Of course, the given treatment is decided by the treating physician and takes into account the specific needs of the patient, conforming to the overall concept and guidelines recommendations.

Even though COPD cannot be cured, optimal management provides symptom relief and control, slows the disease progression, reduces the rate of exacerbations, and consequently improves health‐related quality of life (HRQL) [5]. To this end, in the last few years, there has been a considerable shift in focus on the identification of “early” COPD, which is tied to a better prognosis [6]. Moreover, it is obvious that even the most tailored treatment is ineffective if not taken as prescribed or even at all; therefore, adherence is of great importance and should be assessed in all COPD patients regularly. For this purpose, several methods have been proposed and compared in the literature for measuring adherence [7]. Patient questionnaires and subsequent scorings offer a simple and practical means for assessing adherence in the clinical setting and possibly pinpointing the factors that affect it. Some of the most popular scores are the 8‐item Morisky Medication Adherence Scale (MMAS‐8),^1^ the Test of Adherence to Inhalers (TAIs), and the Adherence to Refills and Medications Scale (ARMS). The MMAS‐8 is a self‐reported medication‐taking behavior scale that is considered the most commonly used self‐reporting method to determine adherence to chronic diseases [8, 9, 10]. TAI is a questionnaire for assessing inhaler adherence in patients with COPD or asthma [11]. ARMS consists of two domains: adherence to taking medications and adherence to refilling prescriptions [12].

Subsequently, the impact of treatment must be assessed and quantified; for this purpose, quality‐of‐life scores have been proposed. Some of the most widely used scores in respiratory diseases and COPD in particular are the St. George's Respiratory Questionnaire (SGRQ)—also in its COPD‐specific version (SGRQ‐C), the Clinical COPD Questionnaire (CCQ), and the COPD Assessment Test (CAT). The SGRQ‐C with 40 items provides three component scores for symptoms, activity, and impact and a total score [13]. The CCQ measures health status and has been used to assess HRQL in COPD and elsewhere [14, 15, 16]. CAT is a relatively simple instrument for quantifying the symptom burden of COPD in routine practice [17].

The relationship between adherence to treatment and quality of life has been studied in various chronic diseases [18] and COPD in particular, with equivocal conclusions. There appears to be a bidirectional relationship [19] where higher adherence leads to better life quality and inversely a patient is more likely to follow a treatment with a positive impact on his overall well‐being. Among the factors affecting treatment adherence, comorbidities play an important role in various manifestations, e.g., polypharmacy and regimen complexity, side effects and interactions among medications, reimbursement issues, etc. [20, 21]. Similarly, the impact of comorbidities on the life quality of COPD patients has been well established [22, 23]. Therefore, COPD‐related studies are often performed with regard to comorbidities that often coexist and affect the overall course of the disease.

In the current observational study, in primary care settings, we enrolled approximately 700 COPD patients (FEV1% predicted < 60% at baseline), with a twofold purpose: (i) to evaluate the adherence to treatment with fluticasone and salmeterol via the Elpenhaler® at 1 and 3 months since treatment initiation, as assessed by MMAS‐8, and (ii) to evaluate the health status and quality of life of the patients, as assessed by the CCQ at 1 and 3 months since treatment initiation. Furthermore, we assessed adherence and health status after stratifying the patients into two groups, namely, with and without comorbidities.

## 2 Materials and Methods

### 2.1 Study Design

This is an open‐label, noninterventional, observational study (NCT03299673) of Greek patients diagnosed with COPD of variable severity.

### 2.2 Setting

Overall, 723 patients were enrolled, out of which 716 patients were eventually included in the statistical analysis. The patients were followed up for 3 months, commencing from December 2017 to July 2018.

### 2.3 Intervention

Written informed consent was obtained from all participants. At the beginning of the study, the patients were initiated on treatment with a fixed‐dose combination (FDC) of fluticasone propionate and salmeterol, 500 + 50 mcg of Rolenium® via the Elpenhaler® device.

### 2.4 Participants

The inclusion criteria were as follows:

1. COPD patients, FEV1% predicted < 60%
2. Compliance with study procedures
3. Newly initiated treatment with combination of fluticasone propionate+salmeterol (500 + 50 mcg) through inhalation Elpenhlaler® device
4. Patients who
  - Are under single LABA bronchodilator
  - Are under single LAMA bronchodilator
  - Are under double LABA/LAMA bronchodilator
  - Have never received or have received ICS‐containing maintenance therapy or systemic corticosteroids on demand, as long as they had not been treated with such medication during the last trimester before study initiation

The exclusion criteria were as follows:

1. History of nonproper use of inhaled treatments
2. Previous ICS or systemic corticosteroids use on‐demand within the last trimester
3. Noncompliance with study procedures

### 2.5 Objectives

The primary objective of the study was to evaluate the adherence to treatment with FDC fluticasone propionate and salmeterol (500 + 50 mcg) via Elpenhaler® at 1 and 3 months since treatment initiation, as assessed by the MMAS‐8 scale, which has been widely used for this purpose in several chronic diseases [24]. This self‐reported scale contains 7 items answered with a yes or no and 1 item with a 5‐point Likert scale, with scores ranging from 0 to 8. The respective MMAS‐8 scores were trichotomized into the following three levels of adherence: high adherence (HA, score = 8), medium adherence (MA, 6 ≤ score<8), and low adherence (LA, score<6) [25].

The secondary objective was to evaluate the health status and quality of life of the patients as assessed by the CCQ at 1 and 3 months (±2 weeks) since treatment initiation. For this study, permission was obtained to use CCQ, which measures health status and is used to assess HRQL. The CCQ consists of ten questions distributed in three domains: (a) symptoms (4 questions), (b) mental state (4 questions), and (c) functional state (2 questions). The questions apply to the previous week and use a 7‐point scale from 0 to 6. The CCQ total score is calculated as the mean of the sum of all items, with a higher value indicating a lower health status. The minimal difference in the CCQ score considered to be of clinical importance is 0.4. Finally, the safety of patients was assessed via adverse event registration throughout the study.

### 2.6 Statistical Analysis

Continuous variables were summarized with the use of descriptive statistical measures (mean value, standard deviation [SD], min, max, median, and IQR). 95% CIs were provided for the mean change in primary and secondary endpoints. Descriptive statistics were also used in summarizing changes between 0 and 1 month, 1 and 3 months, and 0 and 3 months. Categorical variables were displayed as frequency tables (*N*, %). The chi‐square test was used to identify the possible association between categorical parameters. In case of > 2 categorical parameters, the Kruskal–Wallis test was used. All the statistical tests were two‐sided and were performed at a 0.05 significance level. The Kolmogorov–Smirnov test of normality was performed before the assessment of a statistical test. In cases of nonnormality of the data, nonparametric methods were used. Analysis was performed on the basis of nonmissing information, and no imputation methods were applied.

Adherence to treatment with FDC fluticasone propionate and salmeterol (500 + 50 mcg) via Elpenhaler® was assessed by the MMAS‐8 scale. The continuous scale score (0–8) was descriptively summarized at 1 and 3 months, and the change in score between study visits was evaluated by a paired *t*‐test. The MMAS‐8 score was also categorized into HA (score = 8), MA (6 ≤ score < 7), and LA (score < 6). and was further summarized by absolute and relative frequencies (*N*, %). As for the quality of life assessment, the change in the total score of CCQ from treatment initiation at 3 months was evaluated by a paired *t*‐test.

## 3 Results

There were 7 patients excluded from the statistical analysis due to an improper visit schedule. The study population analyzed was equal to 716 patients. The mean length of the study period was 3.02 ± 0.19 months, with 705 patients (98.5%) completing the 3 months of follow‐up.

### 3.1 Demographic and Baseline Characteristics

Most of the patients were men (59.8%), with the majority being 60–74 years old (51.8%). It is observed that only 9.8% of the patients were > 80 years old. The mean body mass index (BMI) of the patients in the study was 28.32 ± 4.34, a rate indicating that the majority of patients had increased body weight. In addition, 44.7% of the study patients were of secondary education, while 16.6% of the patients were of higher education. Regarding the smoking status, 18.3% of the patients were nonsmokers, while 48.6% were current smokers, with a median number of pack‐years being 44.18. Sixty‐three patients (8.8%) discontinued smoking up to 1 month after study initiation, while another one patient discontinued smoking up to the end of the follow‐up period. As for comorbidities, 162 patients (22.6%) had no comorbidities, while the remaining 554 patients (77.4%) had at least one comorbidity. In addition, 512 patients (71.5%) had at least one cardiovascular and/or metabolic disorder, 257 patients (35.9%) had only one cardiovascular disorder, and 200 patients (27.9%) had only one metabolic disorder. Note that only 1.1% of the study patients had diabetes mellitus. Two hundred seventy‐six patients (49.8%) had > 2 comorbidities. Four hundred four out of 554 patients (72.9%) with at least one comorbidity had cardiovascular disease, with metabolic diseases to follow (69.9%). More details are given in Table 1.

**TABLE 1:** Demographic, smoking status, and comorbidities of patients at baseline.

Demographics	Ν (%)
Gender, no. (%)	*Ν* = 716
Male	428 (59.8)
Female	288 (40.2)
Age category, no. (%)	*Ν* = 716
< 50	59 (8.2)
50–65	253 (35.3)
65–80	334 (46.6)
> 80	70 (9.8)
Race, no. (%)	*Ν* = 716
White	716 (100)
Asian	0 (0)
Black	0 (0)
Other	0 (0)
Height (cm)	*Ν* = 716
Mean ± SD	169.61 ± 8.33
Weight (kg)	*Ν* = 716
Mean ± SD	81.47 ± 13.67
BMI (kg/m^2^)	*Ν* = 716
Mean ± SD	28.32 ± 4.34
Education level, no. (%)	*Ν* = 716
Primary	277 (38.7)
Secondary	320 (44.7)
Higher	119 (16.6)

Smoking status	***Ν*** (%)
Smoking status, no. (%)	*Ν* = 716
Nonsmoker	131 (18.3)
Ex‐smoker	237 (33.1)
Time from discontinuation (years)	
Mean ± SD	9.39 ± 6.57
Pack‐years (mean ± SD)	38.57 ± 23.60
Current smoker	348 (48.6)
Pack‐years (mean ± SD)	44.18 ± 22.53
No. (%) of patients who discontinued smoking up to 1 month after study initiation	63 (8.8)
No. (%) of patients who discontinued smoking between 1 and 3 months after study initiation	1 (0.1)

Comorbidities	***Ν*** (%)
No. (%) of patients with no comorbidities	162 (22.6)
No. (%) of patients with at least one comorbidity	554 (77.4)
Number of comorbidities per patient, no. (%)	*N* = 554
1	129 (23.3)
2	149 (26.9)
> 2	276 (49.8)
Number of patients per comorbidity category, no. (%)	
Cardiovascular diseases	404 (72.9)
Metabolic diseases	387 (69.9)
Psychiatric diseases	130 (23.5)
Gastrointestinal diseases	167 (30.1)
Malignancies	16 (2.9)
**Spirometry**	***N*** **= 324**
FEV1% predicted (mean ± SD)	51.50 ± 6.88

According to Table 2, the mean duration of COPD from diagnosis up to study initiation was 8.71 ± 6.40 years. Dyspnoea was assessed using the modified Medical Research Council (mMRC) scale [26], with patients assessing their perceived breathlessness on a scale of 0 (“Breathless with strenuous exercise”) to 4 (“Too breathless to leave the house or breathless when dressing or undressing”). A total of 72% of the patients had a reported dyspnea of Grade 1 or 2, indicating difficulty when walking due to dyspnea. Seven hundred fourteen patients (99.7%) had at least one COPD exacerbation up to 12 months prior to study initiation, with the majority of them (~70%) having 1 or 2 exacerbations. Three hundred ninety‐six patients (55.3%) had at least one past hospitalization the last year prior to study initiation, with 348 patients having only one hospitalization. Moreover, 579 out of 716 patients (80.9%) were vaccinated at least once for influenza or pneumococcus, with 388 patients being vaccinated for both. As for the treatment, detailed data are shown in Table 2; only one patient did not receive any COPD treatment in the past, while the remaining 715 patients (99.9%) received at least one COPD treatment prior to study initiation. The most common COPD treatment received prior to study initiation was LABA (44% of the total number of patients). Moreover, 201 patients (28.1%) received LAMA, which was the second most common COPD treatment. A total of 76.8% of the patients received monotherapy in the past (one active substance), while 23.1% of the patients received double combination therapy (with the most frequent being LABA/LAMA). More details are shown in Table 2.

**TABLE 2:** COPD history, vaccination status, and previous COPD treatment.

COPD history	Ν (%)
**Time from COPD initiation (years)**	
Mean ± SD	8.71 ± 6.40
**mMRC dyspnea scale, no. (%)**	***N* = 714**
Grades 0–1	335 (46.8)
Grades 2–4	379 (52.9)
No. (%) of patients with at least one COPD‐related exacerbation during the last year before study initiation	714 (99.7)
**Number of exacerbations/patient, no. (%)**	
0	2 (0.3)
1	242 (33.8)
2	252 (36.2)
3	120 (16.8)
> 3	93 (12.9)
No. (%) of patients with at least one COPD‐related hospitalization during the last year before study initiation	396 (55.3)
**Number of hospitalizations/patients, no. (%)**	
0	320 (44.6)
1	348 (48.7)
≥ 2	48 (6.7)
**No. (%) of patients with at least one vaccination**	579 (80.9)
Influenza	137 (19.1)
Pneumonococcus	54 (7.5)
Both	388 (54.2)
**Previous COPD treatment**	**(*N* = 716, %)**
No. (%) of patients with no past COPD treatment	1 (0.1)
No. (%) of patients with at least one past COPD treatment	715 (99.9)
Past COPD treatment (active substance), no. (%)	
0	1 (0.1)
1	550 (76.8)
LABA	315 (44)
LAMA	201 (28.1)
LAMA + SABA on demand	34 (4.7)
2	165 (23.1)
LABA/LAMA	122 (17)
LABA/ICS, with different devices (without any treatment at least 3 months prior to study initiation)	12 (1.7)
LABA/ICS, with fixed combination devices (without any treatment at least 3 months prior to study initiation)	31 (4.3)

All study patients received FDC fluticasone propionate and salmeterol (500 + 50 mcg) via Elpenhaler® at baseline. Five hundred eighty‐four patients (81.6%) received only the study drug as monotherapy, while 132 patients (18.4%) received ICS/LABA via Elpenhaler® in combination with other COPD treatments. The most frequently administered combination therapy during the study was ICS + LABA + LAMA (13.5%), i.e., FDC fluticasone propionate and salmeterol (500 + 50 mcg) via Elpenhaler® in combination with LAMA. Only 6.4% of the patients had a change in COPD treatment at 3 months.

### 3.2 Medication Adherence

**FIGURE 1:**
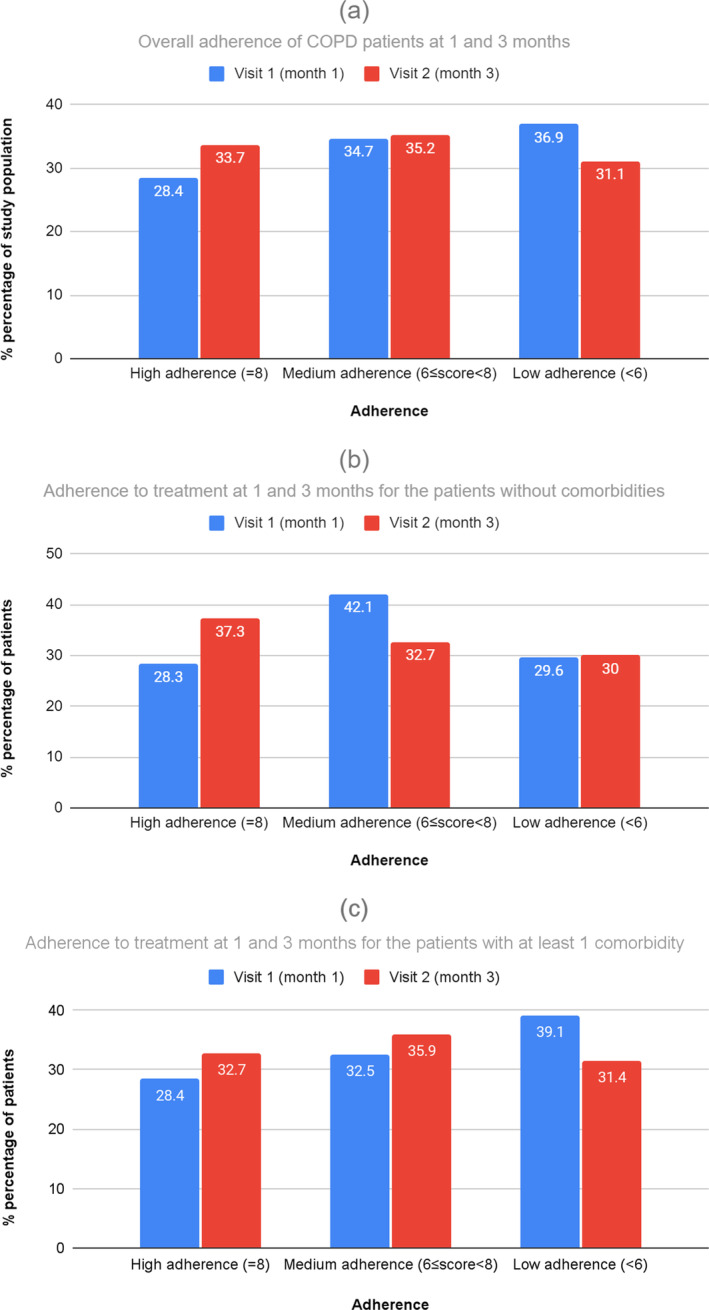
(a) % adherence of COPD patients to treatment with FDC fluticasone propionate and salmeterol (500 + 50 mcg) via Elpenhaler®, as assessed by MMAS‐8 at 1 and 3 months. (b) % adherence to treatment with FDC fluticasone propionate and salmeterol (500 + 50 mcg) via Elpenhaler®, as assessed by MMAS‐8 at 1 and 3 months, for the patients without comorbidities. (c) % adherence to treatment with FDC fluticasone propionate and salmeterol (500 + 50 mcg) via Elpenhaler®, as assessed by MMAS‐8 at 1 and 3 months, for the patients with at least one comorbidity.

The MMAS‐8 is one of the simplest self‐report scales measuring medication adherence behavior. The mean ± SD MMAS‐8 score at 1 and 3 months was 6.12 ± 1.89 and 6.45 ± 1.80, respectively, indicating MA to treatment with FDC fluticasone propionate and salmeterol (500 + 50 mcg) via Elpenhaler® throughout the 3‐month period of its administration. However, there was a statistically significant increase of 0.33 units (95% CI: 0.22, 0.46) in the MMAS‐8 score at 3 months from Visit 1 (Month 1), according to paired *t*‐test (*p* < 0.0001). The MMAS‐8 scores for each patient were also categorized into HA (score = 8), MA (6 ≤ score < 8), and LA (score < 6). As shown in Figure 1a, the majority of patients (> 63%) showed medium to HA (MMAS‐8 score ≥ 6) throughout the study; however, the patients in this category differed significantly between 1 and 3 months (Pearson chi‐square, *p* < 0.0001). More specifically, there were 77 patients changing from low to medium (36 from medium to low) and 21 patients from low to high (15 from high to low) adherence at 3 months. There were also 21 patients changing from medium to high (36 from high to medium) adherence at 3 months.

Figure 1b shows the MMAS‐8 score at 1 and 3 months, as well as the change in the score between visits for the patients without comorbidities. We observe that patients without comorbidities exhibited mostly MA to treatment with FDC fluticasone propionate and salmeterol (500 + 50 mcg) via Elpenhaler® throughout the 3‐month period of its administration. The MMAS‐8 score increased by 0.09 (95% CI: −0.14, 0.32) at 3 months from Visit 1 (Month 1); however, this increase was not statistically significant (*p* = 0.452). Moreover, the majority of the patients without comorbidities (~70%) showed medium to HA (MMAS‐8 score ≥ 6) throughout the study.

Figure 1c depicts the MMAS‐8 score at 1 and 3 months, as well as the change in the total score between visits for the patients with at least 1 comorbidity. Patients with at least one comorbidity exhibit mostly MA to treatment with FDC fluticasone propionate and salmeterol (500 + 50 mcg) via Elpenhaler® throughout the 3‐month period of its administration. Moreover, the number of patients with at least 1 comorbidity that showed medium to HA (MMAS‐8 score ≥ 6) increased from 330 to 363 (increased by 7% from Month 1).

### 3.3 COPD Health Status and Quality of Life

As we can see in Table 3, the majority of the patients at baseline were moderately limited regarding strenuous physical activities, and they often had coughs and phlegm. In all cases, the mean scores of the CCQ questions decreased from 0.63 (short of breath at rest, i.e., from 1.51 ± 1.19 to 0.88 ± 0.79) to 1.46 (strenuous physical activities, i.e., from 2.99 ± 1.44 to 1.53 ± 1.08) at 3 months.

**TABLE 3:** COPD health status as assessed by CCQ questionnaire at 1 and 3 months for the total study population (*N* = 716).

CCQ questions (mean ± SD)	Visit 1 (Month 1) (N = 716)	Visit 2 (Month 3) (N = 683)
*On average, during the past week, how often did you feel*:		
1. Short of breath at rest?	1.51 ± 1.19	0.88 ± 0.79
2. Short of breath doing physical activities?	2.77 ± 1.26	1.52 ± 0.86
3. Concerned about getting a cold or your breathing getting worse?	2.38 ± 1.30	1.30 ± 0.98
4. Depressed (down) because of your breathing problems?	1.99 ± 1.35	1.11 ± 0.92
*In general, during the past week, how much of the time*:		
5. Did you cough?	2.97 ± 1.28	1.59 ± 0.91
6. Did you produce phlegm?	2.88 ± 1.35	1.46 ± 0.97
*On average, during the past week, how limited were you in these activities because of your breathing problems*:		
7. Strenuous activities (such as climbing stairs, hurrying, and doing sports)	2.99 ± 1.44	1.53 ± 1.08
8. Moderate activities (such as walking, housework, and carrying things)	2.12 ± 1.35	1.00 ± 0.97
9. Daily activities at home (such as dressing and washing yourself)	1.34 ± 1.23	0.53 ± 0.76
10. Social activities (such as talking, being with children, and visiting friends/relatives)	1.10 ± 1.19	0.39 ± 0.72
Total CCQ score (mean ± SD)	2.20 ± 1.00	1.13 ± 0.67

**FIGURE 2:**
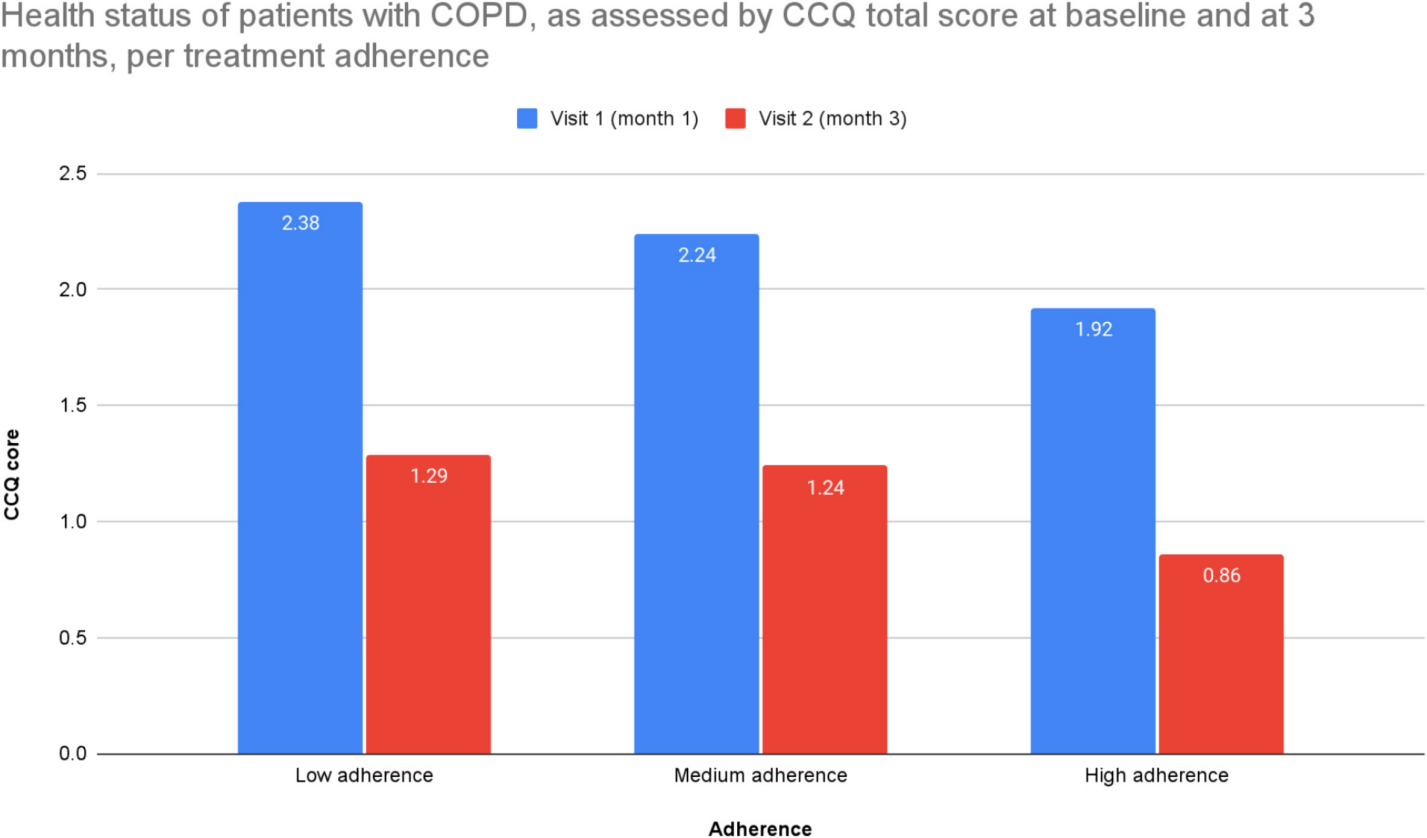
Health status of patients with COPD, as assessed by CCQ total score at baseline and at 3 months, per treatment adherence.

In Figure 2, we present the CCQ scores across the treatment adherence groups at baseline and after 3 months. The mean CCQ total score at baseline was 2.38 ± 0.94, 2.24 ± 1.04, and 1.92 ± 0.97 for the patients with low, medium, and HA at 1 month, respectively, indicating that the quality of life of the COPD patients with HA to treatment was more satisfactory compared to the patients with low or MA. The mean CCQ total score at 3 months was 1.29 ± 0.69, 1.24 ± 0.66, and 0.86 ± 0.57 for the patients with low, medium, and HA at 3 months, respectively, indicating that the quality of life of the COPD patients with HA to treatment was more satisfactory compared to the patients with low or MA. More specifically, the mean CCQ total score at 3 months seemed to be statistically significantly different between the treatment adherence categories at 3 months (Kruskal–Wallis test, *p* < 0.0001).

**FIGURE 3:**
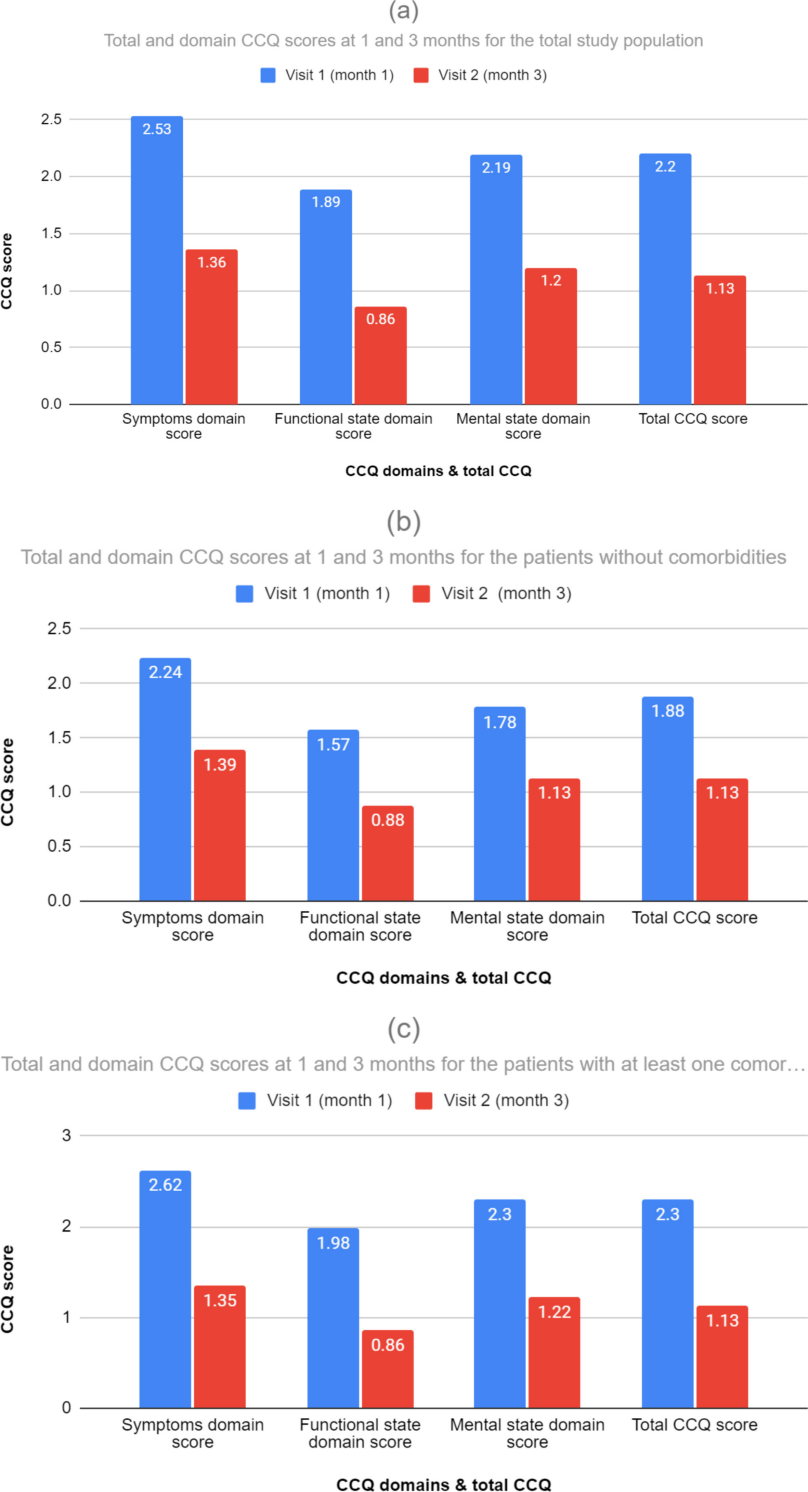
(a) Total and domain CCQ scores at 1 and 3 months and changes from baseline for the total study population. (b) Total and domain CCQ scores at 1 and 3 months and changes from baseline for the patients without comorbidities. (c) Total and domain CCQ scores at 1 and 3 months and changes from baseline for the patients with at least one comorbidity.

Figure 3a shows the total CCQ score and domain scores at baseline and 3 months, as well as the changes in these scores from baseline for the total study population. The mean CCQ total score at 3 months was 1.13 ± 0.67 (starting from 2.2 ± 1.00 at month 1); we observe a statistically significant decrease in the mean CCQ total score by 1.07 (95% CI: −1.17, −1.02; *p* < 0.0001), indicating that the health status of the study patients was improved and quite satisfactory. Similar conclusions were also drawn in the mean domain scores regarding symptoms (score equal to 1.36 ± 0.72), which decreased by 1.18 (95% CI: −1.25, −1.11; *p* < 0.0001), as well as mental and functional state (scores equal to 0.86 ± 0.73 and 1.20 ± 0.88), with a decrease of 1.04 (95% CI: −1.13, −0.96; *p* > 0.0001) and 1.00 (95% CI: −1.10, −0.92; *p* < 0.0001), respectively, at 3 months.

Figure 3b describes the total CCQ score and domain scores at baseline (month 1) and 3 months, as well as the changes in these scores from baseline for the patients without any comorbidities. The mean CCQ total score at 3 months was 1.13 ± 0.63, indicating that the health status of the patients without comorbidities was improved and quite satisfactory (baseline value was 1.88 ± 0.89). More specifically, the mean CCQ total score decreased by 0.75 (95% CI: −0.94, −0.66) at 3 months (*p* < 0.0001). Similar conclusions were also drawn by the statistically significant decrease (*p* < 0.0001) in the mean domain scores regarding symptoms (score equal to 1.39 ± 0.68) with a decrease by 0.86 (95% CI: −1.05, −0.73; *p* < 0.0001), as well as functional and mental state (scores equal to 0.88 ± 0.67 and 1.13 ± 0.86) decreasing by 0.70 (95% CI: −0.90, −0.60; *p* < 0.0001) and 0.65 (95% CI: −0.89, −0.51; *p* < 0.0001), respectively, at 3 months.

Figure 3c depicts the total CCQ score and domain scores at baseline (Month 1) and 3 months, as well as the changes in these scores from baseline for the patients with at least one comorbidity. The mean CCQ total score at 3 months was 1.13 ± 0.68, indicating that the health status of the patients with at least one comorbidity was improved and quite satisfactory (baseline value was 2.30 ± 1.00). More specifically, the mean CCQ total score decreased by 1.18 (95% CI: −1.26, −1.09) at 3 months (*p* < 0.0001). Similar conclusions were also drawn by the statistically significant decrease (*p* < 0.0001) in the mean domain scores regarding symptoms (score equal to 1.35 ± 0.73) with a decrease of 1.27 (95% CI: −1.36, −1.18; *p* < 0.0001), as well as functional and mental state (scores equal to 0.86 ± 0.75 and 1.22 ± 0.88) with a decrease of 1.13 (95% CI: −1.22, −1.03; p < 0.0001) and 1.08 (95% CI: −1.20, −0.99; *p* < 0.0001), respectively, at 3 months.

### 3.4 Adverse Events

Regarding the adverse events, only one patient had an adverse event during the study. Specifically, the patient reported hoarseness, which constituted a nonserious adverse event; nevertheless, the patient discontinued the study treatment.

## 4 Discussion

In the current study, approximately 700 COPD patients were administered a FDC of fluticasone propionate and salmeterol (500 + 50 mcg) through an Elpenhaler® device, according to their primary care treatment physicians' decision. Patients were evaluated at baseline and after 3 months from treatment initiation in terms of medication adherence as well as their health status. We observed a considerable shift of patients during the study's 3‐month follow‐up towards the HA group, denoting, among others, the patients' improved adherence due to the use of FDC fluticasone propionate and salmeterol (500 + 50 mcg) via the Elpenhaler® device. In a similar manner and primarily due to this shift towards higher adherence, we observed a marked improvement in the patients' health status. This tendency was observed in all aspects of the CCQ score (symptoms, functional, and mental state of the COPD patients), which was evident in patients with and without comorbidities.

Based on the subgroup analysis of the CCQ score across the adherence groups both at baseline and after 3 months of treatment administration, we observed an increase in the HA group and subsequently a decrease in the CCQ score, denoting better health status. Since the provided treatment regimen contained additional substances, it is difficult to quantify the contribution of the device to the observed benefit for the patients. Moreover, the healthy adherer effect should be taken into consideration, which often impedes bias when interpreting the association between adherence and quality of life [27]. Patient satisfaction with the inhaler device is one of the factors affecting the adherence of patients to treatment. In this perspective, the role of the Elpenhaler® device has been evaluated in a Greek population diagnosed with COPD and asthma [28, 29]. As far as adherence is concerned, the Elpenhaler® device, when compared with other frequently used inhalation devices, exhibited the lowest error rates for critical errors in the inhalation maneuver [30]. These characteristics of the Elpenhaler® device may represent major reasons for the good adherence observed in this study [31].

We have also assessed the patients' health status as measured by the CCQ score, taking into account their comorbidities. Specifically, we determined the health status of patients with and without comorbidities at baseline and at the end of the follow‐up period. We observed that, as with the total population, the CCQ score is lower both at baseline and after 3 months of treatment, regardless of the comorbidity status. As expected, the initial CCQ score in the subgroup of patients with at least one comorbidity is higher at the beginning of the study (CCQ score = 2.3) compared with the subgroup of patients without any comorbidities (CCQ score = 1.88). Nevertheless, at the end of the study, the total CCQ score for both groups of patients is significantly lower and identical (i.e., CCQ score = 1.13). The same observation can be derived for the individual domain scores comprising the overall CCQ score. At this point, it is worth noticing that the quality of life of Greek patients with COPD has been found to be significantly lower than both that in the general population in Greece and that of patients with COPD reported in other countries [32]. This could be partly attributed to the limited knowledge of Greek COPD patients regarding their disease and subsequent actions for self‐management [33].

According to GOLD 2023 guidelines, ICS + LABA is no longer encouraged, and in cases where ICS is indicated, triple therapy should be preferred [3, 4]. Nevertheless, according to the same report, an ICS combined with a LABA is more effective than the individual components in improving lung function and health status, as well as reducing exacerbations [34]. The impact of treatment with ICS + LABA on exacerbations, in particular, has been tied, according to several studies [35, 36], to the blood eosinophil count, which cannot be reproduced in the current study. There is, however, increasing evidence that blood eosinophil counts are higher in COPD patients [37, 38]. Moreover, the treatment effect of ICS‐containing regimens (including the combination of fluticasone propionate and salmeterol via the Elpenhaler® device) has been found to be more notable in patients with high exacerbation risk [35, 39]. On the other hand, there is also high‐quality evidence denoting the impact of ICS on the airway microbiome and its association with oral candidiasis, hoarse voice, and pneumonia [40].

For the purposes of the current study, we have recruited a relatively large cohort of patients diagnosed with COPD of variable severity from all over Greece. However, featuring patients from a single country and therefore race, generalizations should be made with caution. In the same sense, it should be pointed out that FEV1% predicted < 60% was set as an inclusion criterion, therefore denoting relatively severe COPD patients, further affecting generalizability. Moreover, sample size calculation was not performed, possibly leading to an overpowered study, yet the heterogeneity between study groups (i.e., different sizes of subgroups) was not an issue considering the total study population. Another limitation of the current study is the subjectivity of the methods used for measuring adherence. Even though questionnaires and patient self‐reports comprise simple, inexpensive methods that are readily available in the clinical setting, they are often susceptible to error and subjectivity [7]. Nevertheless, more objective measures, e.g., digital inhalers and other electronic medication monitors, come with a considerable cost and are still not widely available [41]. The Hawthorne effect should also be taken into account, as in most studies, the lack of a control group possibly amplifies its contribution, but it is challenging to assess how much of a factor it is. Overall, since this is not a randomized controlled trial, it is evident that all conclusions gained should be dealt with with caution, and broad generalizations might be susceptible to bias.

## 5 Conclusions

In the current study, we evaluated the treatment adherence of more than 700 COPD patients receiving a fixed combination of fluticasone propionate and salmeterol (500 + 50 mcg) through the Elpenhaler® device. Besides an initial satisfactory adherence, we observed that by the end of the 3‐month follow‐up period, there was a considerable increase in treatment adherence. This observation was further extrapolated and tied to better health status for all enrolled patients, as well as when further subgroupings were considered based on their comorbidity status.

## Author Contributions

K.K. and P.S. conceived and coordinated the study. K.P.E. and K.K. drafted the manuscript. G.H. and A.G. performed the analyses and contributed to the manuscript. P.P. contributed to the interpretation, review, and revision of the manuscript. All authors read and approved the final manuscript.

## Ethics Statement

The study was conducted according to the guidelines of the Declaration of Helsinki and approved by the Institutional Review Board of the two hospitals that participated in the study (“Sotiria” General Hospital of Chest Diseases, Athens, and University General Hospital of Alexandroupolis); reg. no: 10/21.12.2017.

## Consent

Written informed consent was obtained from all subjects involved in the study.

## Data Availability

The data that support the findings of this study are available on request from the corresponding author. The data are not publicly available due to privacy or ethical restrictions.

## Endnote

^1^
The MMAS‐8 Scale (U.S. Copyright Registration No. TX0008632533), content, name, and trademarks are protected by US copyright and trademark laws. Permission for use of the scale and its coding is required. A license agreement is available from MMAR, LLC., www.moriskyscale.com.
